# microRNA strand selection: Unwinding the rules

**DOI:** 10.1002/wrna.1627

**Published:** 2020-09-20

**Authors:** Jeffrey C. Medley, Ganesh Panzade, Anna Y. Zinovyeva

**Affiliations:** ^1^ Division of Biology Kansas State University Manhattan Kansas USA

**Keywords:** arm switching, miR, miR*, miRNA, passenger, strand selection

## Abstract

microRNAs (miRNAs) play a central role in the regulation of gene expression by targeting specific mRNAs for degradation or translational repression. Each miRNA is post‐transcriptionally processed into a duplex comprising two strands. One of the two miRNA strands is selectively loaded into an Argonaute protein to form the miRNA‐Induced Silencing Complex (miRISC) in a process referred to as miRNA strand selection. The other strand is ejected from the complex and is subject to degradation. The target gene specificity of miRISC is determined by sequence complementarity between the Argonaute‐loaded miRNA strand and target mRNA. Each strand of the miRNA duplex has the capacity to be loaded into miRISC and possesses a unique seed sequence. Therefore, miRNA strand selection plays a defining role in dictating the specificity of miRISC toward its targets and provides a mechanism to alter gene expression in a switch‐like fashion. Aberrant strand selection can lead to altered gene regulation by miRISC and is observed in several human diseases including cancer. Previous and emerging data shape the rules governing miRNA strand selection and shed light on how these rules can be circumvented in various physiological and pathological contexts.

This article is categorized under:

RNA Processing > Processing of Small RNAs
Regulatory RNAs/RNAi/Riboswitches > Regulatory RNAs
Regulatory RNAs/RNAi/Riboswitches > Biogenesis of Effector Small RNAs

## Graphical abstract

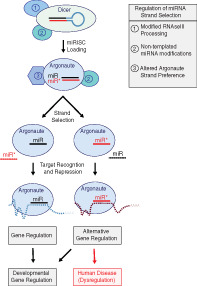

## 1 OVERVIEW OF microRNA BIOGENESIS AND FUNCTION

**FIGURE 1:**
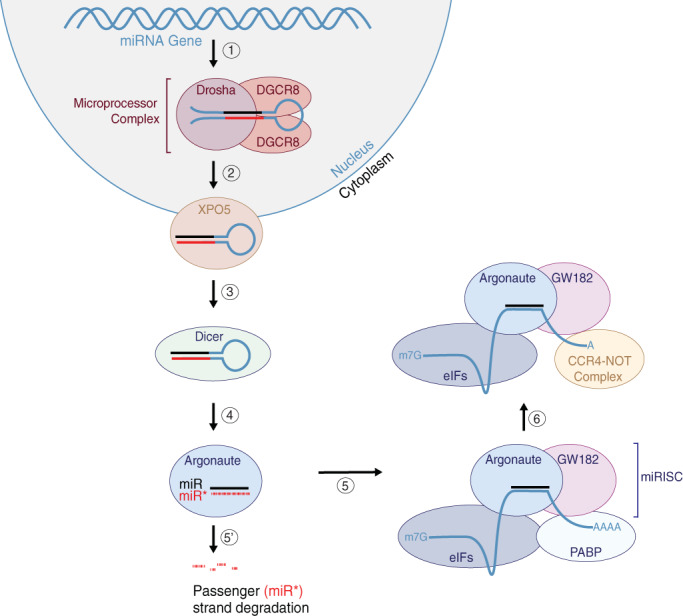
Overview of miRNA biogenesis. (1) Genomically‐encoded miRNAs are transcribed within the nucleus and form a stem‐loop structure that is (2) processed by the Microprocessor complex comprising Drosha and DGCR8/Pasha. (3) The processed miRNA is then transferred to the cytoplasm through Exportin‐5 (XPO5). (4) The stem‐loop precursor miRNA is processed again by Dicer to remove the loop, and the miRNA duplex is loaded into an Argonaute protein. (5) The guide strand of the miRNA duplex is selected for loading into Argonaute to form the miRNA‐induced silencing complex (miRISC), while (5′) the other passenger strand is ejected from the Argonaute and degraded. (6) The miRISC targets mRNAs for silencing based on the seed sequence of the loaded miRNA guide strand

microRNAs (miRNAs) are small, noncoding RNA molecules that control gene expression by regulating the stability or activity of target messenger RNA (mRNA) transcripts (reviewed in Bartel, 2018; Kim, Chang, & Baek, 2017). miRNAs are genomically encoded and transcribed as individual genes or as clusters of several miRNA genes under control of the same promoter (polycistronic) (Kim et al., 2009; Truscott, Islam, & Frolov, 2015). Transcription of miRNA genes produces primary microRNA (pri‐miRNA) transcripts (Lee et al., 2004; Figure 1, Step 1). Within the nucleus, pri‐miRNA transcripts are processed by the Microprocessor complex to form individual stem‐loop miRNA precursors (pre‐miRNAs) that are typically ~70 nucleotides long (Denli, Tops, Plasterk, Ketting, & Hannon, 2004; Gregory et al., 2004; Lee et al., 2003; Lee, Jeon, Lee, Kim, & Kim, 2002; Figure 1, Step 2). The Microprocessor complex comprises the RNAse III‐type endonuclease Drosha and its partner protein DGCR8 (DiGeorge Syndrome Chromosomal Region 8)/Pasha (Denli et al., 2004; Gregory et al., 2004). While the canonical miRNA biogenesis pathway requires processing by the Microprocessor complex, a class of miRNAs referred to as mirtrons are located within introns of protein‐coding genes. Mirtrons have their pre‐miRNA generated directly by splicing and bypass the requirement of processing by the Microprocessor complex (Okamura, Hagen, Duan, Tyler, & Lai, 2007; Ruby, Jan, et al., 2007; Ruby, Stark, et al., 2007). Processed pre‐miRNA molecules are then exported from the nucleus via Exportin‐5 (XPO5) in a Ran‐GTP‐dependent fashion (Lund, Güttinger, Calado, Dahlberg, & Kutay, 2004; Figure 1, Step 3). Upon entry to the cytoplasm, the pre‐miRNA undergoes further processing by a second RNAse III‐type endonuclease, Dicer, which cleaves the loop structure to liberate a miRNA duplex (Grishok et al., 2001; Hutvágner et al., 2001; Jiang et al., 2005; Ketting et al., 2001; Knight & Bass, 2001; Lee et al., 2002; Saito, Ishizuka, Siomi, & Siomi, 2005; Figure 1, Step 4). The miRNA duplex is composed of two RNA strands bound together by Watson‐Crick base pairing. In animals, each strand of the miRNA duplex is typically ~21–22 nucleotides in length with each duplex end containing two nucleotide overhangs typical of RNAse III processing (Creugny, Fender, & Pfeffer, 2018; Han et al., 2004; Schweitz & Ebel, 1971). A distinguishing feature of miRNA duplexes is the presence of bulges and base mismatches. As such, miRNA duplexes are imperfectly paired unlike, for example, small interfering RNAs (siRNAs), which form perfectly paired duplexes.

The miRNA duplex is then loaded into an Argonaute protein to form the miRNA‐Induced Silencing Complex (miRISC; Figure 1, Step 5). One strand of the miRNA duplex is selectively anchored into the Argonaute protein and determines the specificity of the miRISC based on sequence complementarity between the miRNA and the 3′UTR of the target mRNA (Figure 1, Step 5). The miRNA may originate from the 5′ side of the pre‐miRNA (earning it the notation of a “5p” strand) or the 3′ side of the pre‐miRNA (also referred to as “3p” strand). While either strand can be selected for miRISC loading, frequently, a single miRNA strand predominates. For most miRNAs, one strand is preferentially loaded into Argonaute and becomes part of the miRISC, originally earning the designation of the “miR” or “guide” strand. The other strand of the miRNA duplex is ejected from the Argonaute protein and degraded (Figure 1, Step 5′). This typically discarded strand was originally named as “miR*” or “passenger” miRNA. However, in some cases, both arms of the duplex give rise to functional mature miRNAs that are loaded into Argonaute proteins to regulate gene expression (Ghildiyal, Xu, Seitz, Weng, & Zamore, 2009; Okamura, Liu, & Lai, 2009). On such example is miR‐34: in humans, miR‐34b‐5p and miR‐34b‐3p are found at near equal concentrations and target distinct mRNAs (Córdova‐Rivas et al., 2019; Feng, Ge, Du, Zhang, & Liu, 2019). In addition, for majority of miRNAs, both strands have precisely defined 5′ termini, compared to their 3′ ends, suggesting evolutionary pressure to maintain the target‐determining sequence of both strands (Okamura et al., 2008; Ruby, Jan, et al., 2007; Ruby, Stark, et al., 2007; Seitz, Ghildiyal, & Zamore, 2008). This led to a hypothesis that typically less abundant miRNA (miR*) strands might have evolved regulatory functions independently of their respective guide strands (Ruby, Jan, et al., 2007; Ruby, Stark, et al., 2007). Consistent with this idea, the less abundant miR* strands were shown repress mRNAs containing predicted miR* target sites in flies and mammals, suggesting that miR* strands are functional (Okamura et al., 2008; Yang et al., 2010). Such observations have led to widespread use of the 5p/3p nomenclature to define the arm that a miRNA is derived from, acknowledging the functional potential of each miRNA strand. However, because the miRNA guide strand can be derived from either the 5p or the 3p pre‐miRNA arm, it can be difficult to know which miRNA is typically more abundant and which one is degraded. For this reason, we suggest that the miR/miR* or guide/passenger nomenclature remains a convenient method to distinguish, when necessary, between the usually dominant guide (miR) and typically ejected (miR* or passenger) strands. Therefore, we will continue using these terms throughout the review.

The miRISC comprises the miRNA‐loaded Argonaute and an effector protein from the GW182 (Glycine‐Tryptophan Repeat‐Containing Protein of 182 kDa) family that is required for miRISC‐dependent gene silencing (Behm‐Ansmant et al., 2006; Chekulaeva, Filipowicz, & Parker, 2009; Ding & Grosshans, 2009; Ding & Han, 2007; Ding, Spencer, Morita, & Han, 2005; Eulalio et al., 2009; Eulalio, Helms, Fritzsch, Fauser, & Izaurralde, 2009; Eulalio, Huntzinger, & Izaurralde, 2008; Eystathioy et al., 2002; Huntzinger, Braun, Heimstädt, Zekri, & Izaurralde, 2010; Kuzuoglu‐Öztürk, Huntzinger, Schmidt, & Izaurralde, 2012; Lazzaretti, Tournier, & Izaurralde, 2009; Lian et al., 2009; Takimoto, Wakiyama, & Yokoyama, 2009; Till et al., 2007; Tritschler, Huntzinger, & Izaurralde, 2010; Zekri, Huntzinger, Heimstädt, & Izaurralde, 2009; Zhang et al., 2007; Zipprich, Bhattacharyya, Mathys, & Filipowicz, 2009). The GW182 proteins act as molecular scaffolds to bridge Argonaute proteins and downstream effector complexes that mediate miRNA‐dependent translational repression (reviewed in Fabian and Sonenberg, 2012; Jonas & Izaurralde, 2015). A major mechanism for miRNA‐dependent translational repression appears to be inhibition of translation initiation (reviewed in (Bartel, 2018; Gebert & MacRae, 2019; Jonas & Izaurralde, 2015). mRNAs are competent to initiate translation if they have a 5′ methylated cap (m7G cap) and 3′ poly‐A‐tail. Poly‐A‐Binding Proteins (PABPs) associate with the 3′ poly‐A‐tail, whereas eukaryotic initiation factors (eIFs) interact with the 5′ m7G cap. The PABPs and eIFs physically associate with each other to form circular mRNA complexes that are protected from degradation (Derry, Yanagiya, Martineau, & Sonenberg, 2006; Wells, Hillner, Vale, & Sachs, 1998). miRISC has been shown to promote the release of eIF proteins from target mRNAs, which is expected to de‐circularize and destabilize those mRNAs (Fukao et al., 2014; Fukaya, Iwakawa, & Tomari, 2014; Meijer et al., 2013). Further, GW182 physically associates with both Argonaute and PABP and recruits the CAF‐1‐CCR4‐NOT (CAF‐1:Chromatin Assembly Factor 1; CCR4: Carbon Catabolite Repressor 4; NOT: Negative Regulator of Transcription) and PAN‐2‐PAN‐3 (Poly‐A‐specific Ribonuclease) de‐adenylase complexes to miRISC targeted mRNAs (Braun, Huntzinger, Fauser, & Izaurralde, 2011; Christie, Boland, Huntzinger, Weichenrieder, & Izaurralde, 2013; Fabian & Sonenberg, 2012; Gebert & MacRae, 2019; Jonas & Izaurralde, 2015; Kuzuoglu‐Öztürk et al., 2012; Wahle & Winkler, 2013). The cytoplasmic PAN‐2‐PAN‐3 and CCR4‐NOT de‐adenylase complexes act in a consecutive and partially redundant fashion to promote de‐adenylation of miRISC‐targeted mRNAs (reviewed in Bartel, 2018; Jonas & Izaurralde, 2015). The de‐adenylated mRNAs, having reduced poly‐A‐tails, are then de‐capped by DCP2 (mRNA Decapping enzyme) and ultimately targeted for degradation by Xrn1 (exoribonuclease), the primary cytoplasmic 5′‐to‐3′ exoribonuclease (Behm‐Ansmant et al., 2006; Eulalio et al., 2008; Giraldez et al., 2006; Piao, Zhang, Wu, & Belasco, 2010; Rehwinkel, Behm‐Ansmant, Gatfield, & Izaurralde, 2005; Wu, Fan, & Belasco, 2006). However, target mRNA degradation does not appear to be a strict requirement for miRNA‐dependent gene silencing (Bazzini, Lee, & Giraldez, 2012; Freimer, Hu, & Blelloch, 2018). In cell free extracts, de‐adenylated mRNAs are trapped in a translationally repressed state and do not undergo de‐capping or degradation, suggesting that mRNA de‐adenylation may be sufficient for translational repression (Fabian, Sundermeier, & Sonenberg, 2009; Iwasaki, Kawamata, & Tomari, 2009; Wakiyama, Takimoto, Ohara, & Yokoyama, 2007; Zdanowicz et al., 2009). Further, early studies performed in *Caenorhabditis elegans* showed that the *lin‐4* miRNA represses the expression of *lin‐14* and *lin‐28* without affecting the levels of either mRNA (Olsen & Ambros, 1999; Seggerson, Tang, & Moss, 2002). In any case, de‐adenylation is shown to be a wide‐spread outcome of miRISC‐dependent gene regulation and knocking down miRISC components generally leads to increased levels of miRNA targets (Baek et al., 2008; Behm‐Ansmant et al., 2006; Guo, Ingolia, Weissman, & Bartel, 2010; Hendrickson et al., 2009; Selbach et al., 2008). Indeed, mRNA destabilization may play a dominant role in establishing irreversible gene repression (Eichhorn et al., 2014).

While miRNAs typically repress of mRNA targets, there is evidence that some miRNAs may also play nonrepressive roles. During cell cycle arrest, *let‐7* miRNA was shown to promote activation of target mRNAs that are repressed in proliferating cells (Vasudevan, Tong, & Steitz, 2007). Furthermore, RNAs that contain miRNA binding sites can act as sponges, sequestering miRNAs from target mRNAs, and thereby alleviating miRNA‐mediated target repression (reviewed in Thomson & Dinger, 2016). It seems likely that miRNAs may use different mechanisms to regulate mRNA targets, or perhaps the cellular or developmental setting contributes to the regulatory modes of miRISC.

## 2 OVERVIEW OF miRISC LOADING AND miRNA STRAND SELECTION

**FIGURE 2:**
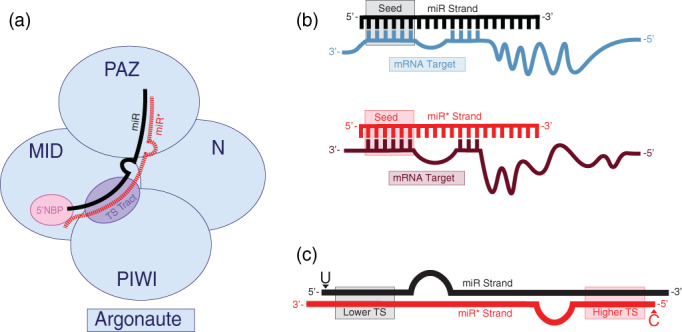
miRISC loading and miRNA strand selection. (a) Schematic representation of Argonaute domains and their relative contributions to miRNA duplex binding and strand selection. The 5′ end of the miRNA guide strand is anchored into a 5′ nucleotide binding pocket (5′NBP) located within the MID domain of Argonaute, which prefers to bind miRNA strands with Uracil at their 5′ ends. The interface of the MID and PIWI domains form a tract that senses the relative TS of each duplex end. The 3′ end of the miRNA guide strand associates with the PAZ domain of Argonaute (modeled after Gerbert and MacRae, 2018). (b) Consequences of miRNA strand selection. (top) The miR strand targets specific mRNAs based on sequence complementarity of the miRNA seed (nucleotides 2–7) to the 3′UTR of target mRNAs. (bottom) As miR* strands contain different seed sequences that their corresponding miR strands, they are expected to target a different set of mRNAs if they are loaded into miRISC. (c) Features associated with guide (miR) and passenger (miR*) strands. In general, the 5′ ends of miRNA guide strands tend to start with Uracil and be situated on the end of the miRNA duplex with lower thermodynamic stability, whereas passenger strands are located on the end of the miRNA duplex with higher thermodynamic stability and tend to start with less favorable nucleotides such as Cytosine (Modeled after Meijer, Smith, & Bushell, 2014)

The Argonaute family of proteins share four functional domains: an N‐terminal domain, PAZ (Piwi/Argonaute/Zwille) domain, MID (middle) domain, and PIWI (P‐element induced wimpy testis) domain (Niaz, 2018; Song, Smith, Hannon, & Joshua‐Tor, 2004; Wang, Sheng, Juranek, Tuschl, & Patel, 2008; Yuan et al., 2005; Figure 2a). Argonautes receive double‐stranded miRNA duplexes to initiate miRISC assembly. The Hsc70‐Hsp90 chaperones (Hsc: Heat‐shock cognate of 71 kDa; Hsp: Heat‐shock protein of 90 kDa) are required for loading of the miRNA duplex into Argonaute in an ATP‐dependent fashion and are believed to promote conformational changes to the Argonaute that allow the miRNA duplex to load (Iwasaki et al., 2009; Nakanishi, 2016; Pare et al., 2009; Tahbaz et al., 2004).

Each strand of the miRNA duplex is destined for different fates. One of the duplexed miRNA strands is selected and anchored into the Argonaute protein, while the other strand is unwound from the complex and is subject to degradation (Figure 2a). The 5′ nucleotide of the Argonaute‐loaded strand is embedded into a binding pocket formed by the interface of the MID and PIWI domains, while the 3′ end resides within a hydrophobic cavity established by the PAZ domain (Boland, Huntzinger, Schmidt, Izaurralde, & Weichenrieder, 2011; Frank, Sonenberg, & Nagar, 2010; Ma et al., 2005; Ma, Ye, & Patel, 2004; Park et al., 2019; Song et al., 2004; Wang et al., 2008; Wang et al., 2009; Yuan et al., 2005) (Figure 2a). While loading of the miRNA duplex into Argonaute is an active process that requires ATP, unwinding of miRNA duplexes occurs in a passive, ATP‐independent fashion (Yoda et al., 2009). The N‐terminal domain of Argonaute is proposed to undergo conformational changes upon binding to the miRNA duplex that promotes passive wedging and release of the strand destined for degradation (Kwak & Tomari, 2012).

The Argonaute‐loaded miRNA programs the gene target specificity of the miRISC, as nucleotides 2‐7, referred to as the miRNA “seed” sequence, are mainly responsible for determining the target mRNA identity through base pairing (Grün, Wang, Langenberger, Gunsalus, & Rajewsky, 2005; Haley & Zamore, 2004; Jackson et al., 2003; Krek et al., 2005; Lewis, Burge, & Bartel, 2005; Lewis, Shih, Jones‐Rhoades, Bartel, & Burge, 2003; Lim et al., 2005; Rajewsky & Socci, 2004; Figure 2b). Additional pairing between nucleotides in the 3′ half of the miRNA and the target mRNA also contribute to miRNA‐mediated gene targeting (reviewed in Chipman & Pasquinelli, 2019). miRNAs that share a common seed sequence, referred to as miRNA families, can target the same group of mRNA substrates, at least in a partially redundant manner. As the miRNA strand selected for Argonaute loading directly determines the mRNA targets, altered strand selection can direct miRISC to repress a different set of target genes (Figure 2b).

How does Argonaute determine which strand of the miRNA duplex should be loaded? Major factors contributing to miRNA strand selection are two intrinsic features of the miRNA duplex: the identities of the 5′ nucleotide (nt) of each strand and the relative thermodynamic stability (TS) of the two ends of the duplex (Figure 2c). Structural analysis of human Ago2, an Argonaute associated with miRNAs, revealed that the 5′ nucleotide binding pocket shows a strong preference for Uracil (Frank et al., 2010. in vitro titration experiments found that the MID domain of human Ago2, a major miRNA‐associated Argonaute, binds to UMP twice as strongly as AMP and approximately 30 times more strongly than CMP or GMP (Frank et al., 2010). Consistently, the most commonly observed 5′ nucleotide of miRNA guide strands is a Uracil, whereas the passenger strand often begins with a Cytosine at the 5′ end (Ghildiyal et al., 2009; Mi et al., 2008; Takeda, Iwasaki, Watanabe, Utsumi, & Watanabe, 2008; Warf, Johnson, & Bass, 2011; Figure 2c). Furthermore, the phosphate moiety of the 5′ nucleotide of the guide strand, but not the passenger strand, is required for miRISC loading of the miRNA guide strand in fly embryonic lysates (Kawamata, Yoda, & Tomari, 2011). Selectively blocking the phosphorylation of the 5′ end of the guide strand in vitro leads to the loading of the passenger strand, consistent with either strand having the capacity to be loaded into miRISC (Kawamata et al., 2011). In addition to favoring a 5′ mono‐phosphorylated Uracil, Argonaute also appears to favor loading the end of the duplex that contains the lower relative thermodynamic stability. The first four nucleotides of each end of the duplex are believed to establish thermodynamic asymmetry, and the difference of a single extra hydrogen bond is sufficient to drive preferential loading of the less stable strand in vitro (Khvorova, Reynolds, & Jayasena, 2003; Krol et al., 2004; Schwarz et al., 2003). Consistently, the 5′ ends of guide strands have been found to contain an excess of purines, whereas the 5′ ends of passenger strands are pyrimidine rich in humans and flies, which likely contributes to thermodynamic asymmetry between the two duplex ends (Hu et al., 2009).

Perhaps not surprisingly, Argonaute protein appears to play a direct role in miRNA strand selection, as recombinant Argonaute MID domain is sufficient to recognize the preferred 5′ nucleotide and to favor thermodynamically unstable ends (Suzuki et al., 2015). Residues within the Argonaute PIWI domain contribute to a phosphate binding tract that is formed along the interface of the MID and PIWI domains that is proposed to sense TS while a nucleotide selection loop within the MID domain senses the identity of the 5′ nucleotide (Suzuki et al., 2015; Figure 2a). Consistently, in *C. elegans*, single amino acid substitutions within the MID (G553R) and PIWI (G716E, S895F, and S939F) domains of the miRNA Argonaute ALG‐1 (Argonaute‐like Gene) reverse the strand selection of certain miRNAs (Zinovyeva, Bouasker, Simard, Hammell, & Ambros, 2014; Zinovyeva, Veksler‐Lublinsky, Vashisht, Wohlschlegel, & Ambros, 2015). None of the ALG‐1 mutations that affect strand selection are located within the guide RNA‐binding pocket and appear to be located toward the exterior surface of the MID or PIWI domains (Zinovyeva et al., 2014). Interestingly, these ALG‐1 mutations do not reverse strand selection for all miRNAs, suggesting that additional intrinsic or extrinsic factors may affect *C. elegans* miRNA strand selection differently for distinct miRNAs (Zinovyeva et al., 2015). Obvious miRNA duplex‐intrinsic factors, such as 5′ nt identity or end stability, were not able to distinguish the strand switching‐miRNAs from miRNAs resistant to ALG‐1 mutations (Zinovyeva et al., 2015). miRNA duplexes also contain mismatches; however, the presence and positions of central mismatches do not appear to play a substantial role in miRNA strand selection in human cells (Suzuki et al., 2015). Overall, Argonaute may directly sense the two major drivers of miRNA strand selection: thermodynamic end stability and 5′ nucleotide identity. It is important to note that much of the data used to formulate the rules of miRNA strand selection were derived from in vitro experiments. While often supported by in vivo observations, such as the observation that most miRNAs begin with a 5′ Uracil (Ghildiyal et al., 2009; Mi et al., 2008; Takeda et al., 2008; Warf et al., 2011), how well these rules hold up in a living organism throughout its lifespan is yet to be established.

## 3 HOW WELL DO miRNAs FOLLOW THE RULES?

**FIGURE 3:**
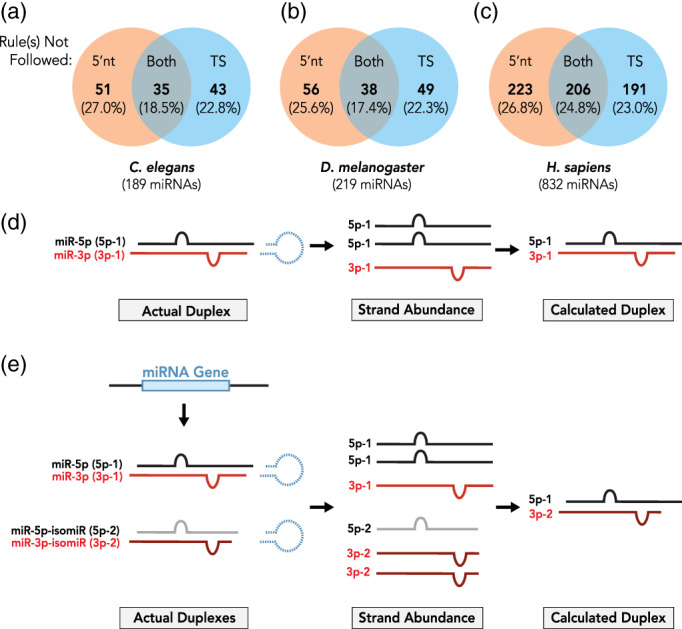
Not all miRNAs follow the rules. Shown are the percentages of miRNAs that do not follow the 5′ nucleotide selection rule, thermodynamic duplex‐end stability rule, or either rule in (a) *Caenorhabditis elegans* (*n* = 189 miRNAs), (b) *Drosophila melanogaster* (*n* = 219), and (c) humans (*n* = 832). miRNA sequences were obtained from miRBase (Release 22.1) and analyzed for duplex formation using RNAduplex (ViennaRNA package v2.4.14) with default parameters. The RNA secondary structure represented in dot‐matrix format was first examined for unpaired, overhanging bases at each duplex end. To determine duplex‐end stabilities, four terminal base pairs at each duplex end were loaded into RNAduplex for terminal minimum free energy (MFE) calculation. ΔΔG for duplex ends was calculated as follows: ΔΔG = Δguide − Δpassenger, where Δguide was the four base‐pair MFE value for the 5′ end of the guide miRNA and Δpassenger was the four base‐pair MFE for the 5′ end of the miRNA*. miRNAs were not considered to follow the 5′ nucleotide selection rule if the 5′ nucleotide of the passenger strand was identical to or more favorable than the 5′ nucleotide of the guide strand. The preferred 5′ nucleotide was defined as U > A > C > G (Frank et al., 2010). miRNAs with equal or higher thermodynamic stability on the guide end of the duplex compared to the passenger end of the duplex were not considered to follow the thermodynamic stability rule. (d) miRNA reads observed in small RNAseq experiments (“Strand abundance”) are thought to originate from the same duplex. (e) Hypothetically, for any given miRNA, miRNA reads observed in small RNAseq experiments may originate from distinct duplexes generated through alternative processing

While the 5′‐nucleotide preference and TS rules are considered to be the primary factors driving miRNA strand selection during Argonaute loading, strand selection of all miRNAs may not be explained by these two rules only. To address how well miRNAs follow the rules of strand selection, we compiled the 5′ nucleotide identity and relative duplex‐end TS of miRNAs from *C. elegans*, *Drosophila*, and humans, using data from the miRNA depository miRBase (Kozomara, Birgaoanu, & Griffiths‐Jones, 2018). We assembled miRNA duplexes based on the most abundant read sequence available for the 5p and 3p strand of each miRNA, using RNAduplex (Lorenz et al., 2011; Lorenz, Hofacker, & Stadler, 2016; Mathews et al., 2004; Turner & Mathews, 2009). miRNAs with extremely low abundance were excluded from our compilation, as were miRNAs where we could not unambiguously determine arm preference. Using RNAduplex from ViennaRNA package v2.4.14 (Lorenz et al., 2011, 2016; Mathews et al., 2004; Turner & Mathews, 2009), we examined the relative TS of the terminal four nucleotide pairs that have been previously determined as sufficient to establish thermodynamic asymmetry on each end of the miRNA duplexes (Schwarz et al., 2003). We also compared the identity of the 5′ nucleotide for the dominant (guide) and less abundant (passenger) strands. The preference of 5′ nt was defined as U > A > C > G (Frank et al., 2010). We considered that a miRNA duplex did not follow the TS rule if the duplex end correlating with the dominant strand's 5′ end had a higher or equal free energy than the duplex end producing the passenger strand. Likewise, if the passenger strand contained an equally preferred or more preferred 5′ nt compared to the dominant strand, we considered that strand selection of that miRNA could not be explained by the 5′ nt rule. In each organism that we examined, half of all miRNAs did not follow one of the two rules for miRNA strand selection (Figure 3a–c). Strikingly, 17–25% of miRNA duplexes did not follow either the 5′ nt preference or TS rules (Figures 3a–c). For these miRNAs, if the 5′ nucleotide and the TS rules were the only determinants of strand selection, we would expect the miR* strand to be selected over the miR strand. However, this analysis carries an assumption that the most abundant 5p and 3p miRNA strands originate from the same duplex (Figure 3d). We cannot exclude the possibility that the miRNA reads that represent each miRNA strand, as reported by miRBase, in fact originate from distinct duplexes (Figure 3e). Overall, miRNA duplexes could be composed of different 5p/3p strands in vivo, with the “unselected” strands degraded and therefore undetected by small RNAseq experiments (Figure 3e). In addition, miRBase‐annotated miRNA sequences or abundances may not be able to accurately reflect tissue‐dependent expression of miRNAs, making it difficult to define a single miRNA duplex from which any given miRNA strand originates. However, the large population of miRNAs where relative strand abundance cannot be explained by either rule suggests that alternative mechanisms may regulate or contribute to strand selection of those miRNAs.

## 4 REGULATION OF miRNA STRAND SELECTION

While the miRNA guide strand is usually observed in extreme excess to its corresponding passenger strand, growing evidence supports that the relative levels of the two strands are highly regulated. An early observation in mice found that the relative expression of 5p and 3p strands varied for certain miRNA duplexes across different tissues (Ro, Park, Young, Sanders, & Yan, 2007). Notably, the miR‐194‐3p strand was nearly undetectable in brain and stomach tissues but was expressed at least as strongly as the miR‐194‐5p strand in other tissues including lungs, ovaries and uteri (Ro et al., 2007). Similar observations have been reported in flies, where several miRNA duplexes were found to exhibit tissue‐specific differences in miRNA strand selection (Ghildiyal et al., 2009). In fly ovaries, the miR‐92a‐3p, miR‐988‐3p, and miR‐284‐5p strands are expressed at higher levels than their corresponding passenger strands, while the miR‐92a‐5p, miR‐988‐5p, and miR‐284‐3p strands are expressed in excess within fly heads (Ghildiyal et al., 2009). This phenomenon of alternative miRNA strand usage is often referred to as miRNA “arm switching”. Interestingly, miRNA arm switching has been proposed to influence miRNA evolution, as altered selection of a miRNA strand by Argonaute would be expected to result in a different mRNA target repertoire (de Wit, Linsen, Cuppen, & Berezikov, 2009; Griffiths‐Jones, Hui, Marco, & Ronshaugen, 2011; Marco, Hui, Ronshaugen, & Griffiths‐Jones, 2010). Interestingly, in some closely‐related species, the preferred miRNA arm for homologous miRNA genes is not the same (de Wit et al., 2009; Griffiths‐Jones et al., 2011; Marco et al., 2010), consistent with the idea that, in at least some cases, miRNA arm switching could be evolutionarily favored.

However, we note that miRNA arm switching may not always be due to changes in miRNA strand selection per se. It has been suggested that the ratio of miR::miR* strands may be influenced by target availability, as the presence of suitable mRNA targets can stabilize cognate miRNAs (Chatterjee, Fasler, Büssing, & Großhans, 2011; Tsai et al., 2016). Therefore, cells expressing mRNAs with miR* target sites may lead to stabilization of miR* strands, resulting in the 5p/3p ratio change without altering the preference of Argonaute for selecting the miR strand. Further, depletion or reduction of the 5′ ➔ 3′ exonucleases *xrn‐1* and *xrn‐2* result in accumulation of specific miRNAs and miR* strands (Chatterjee et al., 2011). Interestingly, this effect is not equal—reduction of *xrn‐1* or *xrn‐2* have been shown to affect the 5p/3p ratios of certain miRNAs (Chatterjee et al., 2011; Miki, Rüegger, Gaidatzis, Stadler, & Großhans, 2014). This observation, combined with the ability of targets to stabilize specific miR* strands, prompted the authors to suggest that XRN‐1/2‐mediated degradation preferentially affects miR* strands capable of Argonaute loading leading to changes in 5p/3p ratios (Chatterjee et al., 2011). Complicating the matters of miRNA strand stability being dictated by cognate target presence, numerous groups have reported target‐directed miRNA degradation (TDMD), a mechanism for target dependent miRNA decay (reviewed in Wightman, Giono, Fededa, & de la Mata, 2018). Under TDMD, the presence of a target mRNA promotes cognate miRNA degradation, often with specific target mRNAs responsible for controlling miRNA decay (Ameres et al., 2010; Cazalla, Yario, Steitz, & Steitz, 2010; de la Mata et al., 2015; Ghini et al., 2018; Kleaveland, Shi, Stefano, & Bartel, 2018). TDMD may promote dissociation of the 3′ end of the miRNA from the Argonaute protein, thereby exposing the 3′ end to modifications that lead to miRNA degradation (Sheu‐Gruttadauria et al., 2019; Yang et al., 2020). Thus, the presence of “decay targets” may in theory influence the 5p/3p ratios of certain miRNAs through TDMD. It is also possible that circRNAs, which have been proposed to act as miRNA sponges (reviewed in Kristensen et al., 2019; Thomson & Dinger, 2016), could influence miRNA arm availability by sequestering miRNAs that have target sites within the circRNA. While sequestering miRNA arms might not affect the 5p/3p ratio of a given miRNA, it could alter the balance of target repression by each miRNA arm. We may speculate further that some mRNA targets or circRNAs could increase miRNA stability, while others could lead to cognate miRNA degradation, allowing for complex effects on miRNA‐mediated gene expression regulation. It is important to determine whether changes to 5p/3p ratios of miRNAs are due to altered miRNA strand selection or alternative mechanisms. As changes in miRNA strand selection would directly influence Argonaute loading, examining the relative abundance of miRNA strands loaded into Argonaute proteins is a critical first step to experimentally validate whether miRNA strand selection is affected under different conditions.

Additional insights into how miRNA strand selection may be regulated come from studies of chemically modified small RNA duplexes that affect strand selection. Introducing mismatches into siRNA duplexes at certain positions of the passenger strand was found to further drive selection of the guide strand (Wu et al., 2011). In particular, chemical modifications that affect the 5′ end stability of the siRNA duplex can affect the relative TS of each end and lead to a reversal in siRNA strand selection (Bramsen et al., 2009; Bramsen & Kjems, 2013; Ui‐Tei, Nishi, Takahashi, & Nagasawa, 2012). Additional modifications that modify the ribose sugar group or substitution modifications to the 2′ hydroxyl group of the ribose can also affect TS of the siRNA duplex and influence strand selection. Further, modifications to the 3′ overhangs can also directly affect TS and can influence strand selection (Bramsen et al., 2009; Bramsen & Kjems, 2013; Ui‐Tei et al., 2012). While the nature of these modifications in miRNA strand selection have not been fully explored, such alterations could presumably influence the TS of all small RNA duplexes and subsequently affect strand selection. Finally, while the physiological significance of these mutations is unclear, collectively, these findings support the notion that, in principle, regulatory modifications to miRNA duplexes may play some role in miRNA strand selection.

If 5′ nucleotide identity and relative duplex‐end stability are the primary factors influencing miRNA strand selection, how might miRNA arm switching occur in developmental or tissue‐specific contexts? There are several possibilities, which include regulatory factors that remodel the miRNA duplex or influence Argonaute strand loading preference in a context‐dependent fashion. Below, we will discuss the roles that altered intrinsic miRNA features and extrinsic protein factors may play in miRNA strand selection regulation.

**FIGURE 4:**
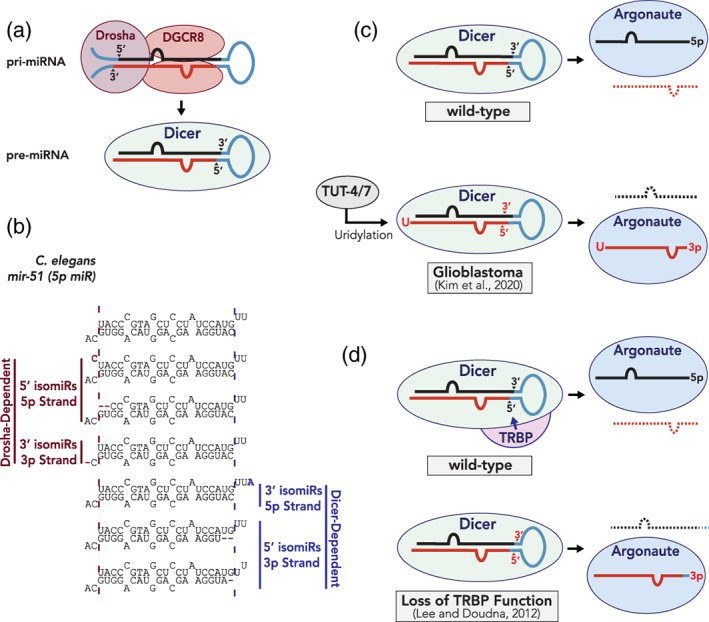
Regulation of miRNA strand selection. (a) Sequential processing of miRNAs by the Microprocessor complex (top) and Dicer (bottom) generates a miRNA duplex. (b) Altered miRNA processing leads to the production of templated isomiRs and may have changes in 5′ nucleotide identity or relative duplex‐end stabilities. *C. elegans* miR‐51 isomiRs are shown as an example. (c) Contribution of uridylation to strand selection of miR‐324 (Kim et al., 2020). In wild‐type tissues expressing low levels of terminal 3′ uridylases (TUTases), nonuridylated miR‐324‐5p is selected for miRISC loading. In Glioblastoma, high expression of TUTases leads to uridylation of miR‐324‐3p, altered Dicer specificity and reversed strand selection toward miR‐324‐5p. (d) Contribution of TRBP to miRNA strand selection. TRBP is proposed to maintain the fidelity of Dicer processing (Lee & Doudna, 2012). In the absence of TRBP, Dicer generates isomiRs, which may lead to altered duplex characteristics that influence strand selection

The same miRNA precursor can produce distinct miRNA isoforms, collectively known as isomiRs. The expression of isomiRs appears to be regulated in a developmental and tissue‐specific manner (Fernández‐Pérez, Brieño‐Enríquez, Isoler‐Alcaraz, Larriba, & del Mazo, 2017; Fernandez‐Valverde, Taft, & Mattick, 2010; Woldemariam et al., 2019; Wyman et al., 2011). miRNA processing by Drosha or Dicer at alternate cut sites results in isomiRs that match the genomically‐encoded pre‐miRNA sequence and are referred to as “templated isomiRs” (Figure 4a,b). These isomiRs can contain truncations or additions to the miRNA duplex and can alter the 5′ nucleotide identity of either, or both, strands. Even a single base truncation or extension on the 5′ end of a miRNA strand can change the identity of the 5′ nucleotide of that strand. Such alteration can remove a preferred 5′ nucleotide from a miR strand or add a favored 5′ nucleotide to a miR* strand, thereby shifting strand selection in favor of the miR* strand (Figure 4b). In addition, these variations can also influence the relative TS of miRNA duplex ends (Lee & Doudna, 2012; Starega‐Roslan, Koscianska, Kozlowski, & Krzyzosiak, 2011). For example, increasing the duplex‐end stability of the 5′ miRNA end or decreasing the duplex‐end stability of the 3′ end may now favor duplex unwinding from the 5′ end of the typically less abundant miR strand, resulting in duplex loading in the opposite orientation (Figure 4b). Indeed, isomiRs resulting from altered Dicer processing sites have been shown to change the thermodynamic properties of mammalian miRNAs, leading to reversed strand selection (Lee & Doudna, 2012; Wilson & Doudna, 2014). Furthermore, most miRNA duplexes contain two nucleotide overhangs at their 3′ ends. Nucleotide compositions of the overhangs have been reported to affect TS of miRNA duplexes in vitro (Miller, Jones, Giovannitti, Piper, & Serra, 2008; O'Toole, Miller, Haines, Zink, & Serra, 2006), therefore altered nucleotide compositions of the overhangs may potentially alter strand selection.

In addition to the hypothesized role of templated isomiRs, nontemplated isomiRs play a role in miRNA strand selection. Nontemplated isomiRs include polymorphisms or nucleotide additions that do not match the genomic pre‐miRNA sequence. One major example is the nontemplated addition of uracil to the 3′ end of a miRNA strand that is catalyzed by 3′ terminal uridyl transferase (TUT) enzymes (reviewed in Menezes, Balzeau, & Hagan, 2018; Pirouz, Ebrahimi, & Gregory, 2019). TUT4 and TUT7, which uridylate *let‐7* in an evolutionarily conserved manner (Chang, Triboulet, Thornton, & Gregory, 2013; Hagan, Piskounova, & Gregory, 2009; Heo et al., 2009; Lehrbach et al., 2009; Thornton, Chang, Piskounova, & Gregory, 2012; Wynsberghe et al., 2011), have recently been shown to affect strand selection of human miR‐324 (Kim et al., 2020). TUT4/TUT7 uridylate the 3′ end of miR‐324‐3p, which changes the site of Dicer processing to generate a shorter miR‐324 duplex that undergoes reversed strand selection, stabilizing the miR‐324‐3p strand instead of the typically more abundant miR‐324‐5p strand (Figure 4c; Kim et al., 2020). Interestingly, TUT4/TUT7 expression levels appear to be regulated in developmental and tissue‐specific fashions, suggesting that regulation of TUT4/TUT7 activity can dictate strand selection of miR‐324 in humans, resulting in arm switching across tissues (Kim et al., 2020).

Another modification to the miRNA duplex is deamination of adenine to inosine (A‐to‐I editing). A‐to‐I editing has been shown to influence miRNA strand selection in mice and human cells (Iizasa et al., 2010; Li et al., 2017). Specifically, mice brain tissue lacking Adenosine Deaminases Acting on RNA (ADAR, *Adarb1* knockout) enzymes exhibit reversed strand selection for some of the miRNAs (Li et al., 2017). Interestingly, A‐to‐I editing events are barely detectable in the mouse early embryo (Li et al., 2017) suggesting that A‐to‐I editing might regulate strand selection in a developmental and/or tissue specific manner.

Additional accessory factors may also play a role in miRNA strand selection, most likely through isomiR formation. In flies, the complex made up of Dcr‐2 and R2D2 (protein containing two dsRNA Binding Domains Associated with Dcr‐2) was proposed to sense the relative TS of perfectly‐paired siRNA duplexes with R2D2 binding to the more stable end thereby passing the less stable end to Ago2, suggesting a direct role of Dcr‐2/R2D2 in strand selection (Tomari, Matranga, Haley, Martinez, & Zamore, 2004). However, miRNA guide‐strand selection appears to occur independently of R2D2 as miRNA guide strand loading is not perturbed by loss of R2D2 function (Nishida et al., 2013; Okamura, Robine, Liu, Liu, & Lai, 2010). Furthermore, mammalian Dicer is dispensable for asymmetric miRNA strand selection in vitro (Betancur & Tomari, 2011; Murchison, Partridge, Tam, Cheloufi, & Hannon, 2005) and fly Dcr‐1 is not required for incorporation of miRNA::miRNA* duplexes into Ago1/miRISC (Kawamata, Seitz, & Tomari, 2009; Yoda et al., 2009). Similarly, the PAZ domain of Argonaute was proposed to receive less stable ends from the Argonaute co‐factors TRBP (TAR RNA‐binding Protein) and PACT (Protein activator of the interferon‐induced protein kinase) (Gredell, Dittmer, Wu, Chan, & Walton, 2010; Noland, Ma, & Doudna, 2011); however, deletion of the PAZ domain did not alter miRNA loading, suggesting PAZ domain is not required for sensing TS (Suzuki et al., 2015). Thus, there are conflicting reports of whether Dicer plays a direct role in miRNA strand selection. One possible explanation is that depletion of TRBP affects the accuracy of Dicer processing and therefore isomiR distribution, which could subsequently alter strand selection. Indeed, changes in Dicer processing sites produce isomiRs with altered thermodynamic end stabilities that reverse strand selection of mammalian miRNAs in vitro (Kim et al., 2014; Lee & Doudna, 2012; Wilson & Doudna, 2014; Figure 4d).

**FIGURE 5:**
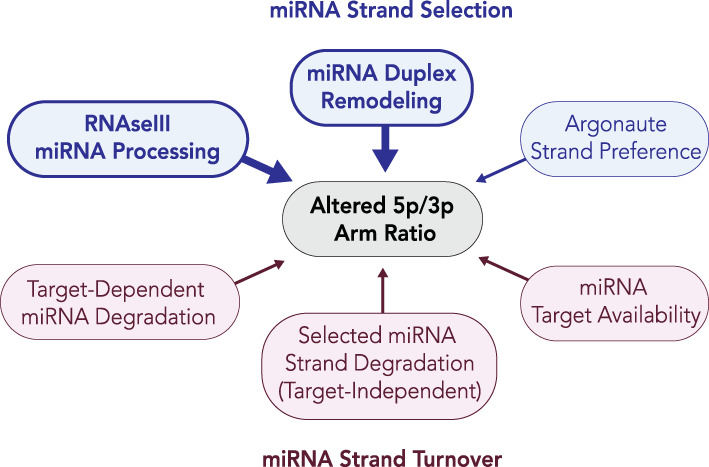
Potential mechanisms influencing miRNA 5p/3p arm ratios. The two mechanisms that may affect the relative abundance of miRNA arms are strand selection and miRNA turnover. We propose that strand selection is likely the dominant mechanism leading to altered arm ratios, as miRNA turnover would be expected to influence miRNA arm ratios after a dominant miRNA strand is selected. Mechanisms influencing the production of isomiRs (highlighted in bold) have been experimentally demonstrated to influence 5p/3p ratios of miRNAs and can lead to miRNA arm switching, whereas other mechanisms remain largely hypothetical

Presumably, any proteins that can regulate the formation of nontemplated isomiRs, including TUTases, adenylases, and ADARs, may do so by either changing the Dicer cut site on the miRNA duplex (Kim et al., 2020; Lee & Doudna, 2012) or by changing the thermodynamic properties of the miRNA duplex and ultimately influence miRNA strand selection. Since isomiRs can also be generated through altered Drosha cut sites, proteins that influence the integrity of Drosha processing could also play a role in strand selection. Indeed, Drosha and Dicer appear to generate 5′ and 3′ isomiRs at similar frequencies (Seitz et al., 2008), although specific examples of Drosha‐generated isomiRs that directly influence miRNA strand selection have been experimentally demonstrated. Overall, isomiRs may be a dominant mechanism driving miRNA strand selection, or arm switching, although it remains possible that unidentified regulators play substantial roles. It is intriguing to speculate that factors interacting with Argonaute could influence its strand preference. Such factors may induce conformational changes in Argonaute that alter the strand preference. Interestingly, Argonaute phosphorylation within its 5′ nucleotide binding pocket influences its ability to bind the guide strands of several miRNAs (Rüdel et al., 2010), and several reports have highlighted roles of Argonaute phosphorylation in miRISC‐dependent gene silencing (Golden et al., 2017; Horman et al., 2013; Huberdeau et al., 2017; Rajgor, Sanderson, Amici, Collingridge, & Hanley, 2018). Although it has not been directly demonstrated, it would be interesting to see whether Argonaute post‐translational modifications, including phosphorylation, play a role in miRNA strand selection. Overall, several mechanisms may contribute to altered miRNA 5p/3p ratios with regulation of miRNA strand selection likely playing a dominant role (Figure 5).

## 5 ALTERNATIVE miRNA STRAND USAGE IN HUMAN DISEASE

The ability of cells to switch the miRNA arm preference is also evident from cases of physiological or pathogenic states, further supporting the idea that miRNA strand selection is a regulated process. Environmental factors have also been observed to influence miRNA strand selection. In human cells, DNA damage has been shown to promote changes in the expression of miRNAs (Dickey, Zemp, Martin, & Kovalchuk, 2011), and there is some evidence suggesting that DNA damage influences strand selection of certain miRNAs (Tarasov et al., 2016). In another example, *Drosophila* ethanol exposure leads to the accumulation of certain miR passenger strands (Ghezzi, Zomeno, Pietrzykowski, & Atkinson, 2016). As ethanol exposure promotes changes in the 5p/3p ratios of certain miRNAs, it is possible that de‐regulation of miRNA strand selection could play some role in alcohol abuse disorders (Ghezzi et al., 2016). However, additional work is required to determine whether these altered 5p/3p ratios result from changes in miRNA strand selection. While changes in miRNA strand abundance can result from several different mechanisms, we propose that altered miRNA strand selection is likely a major mechanism leading to miRNA arm switching (Figure 5).

**FIGURE 6:**
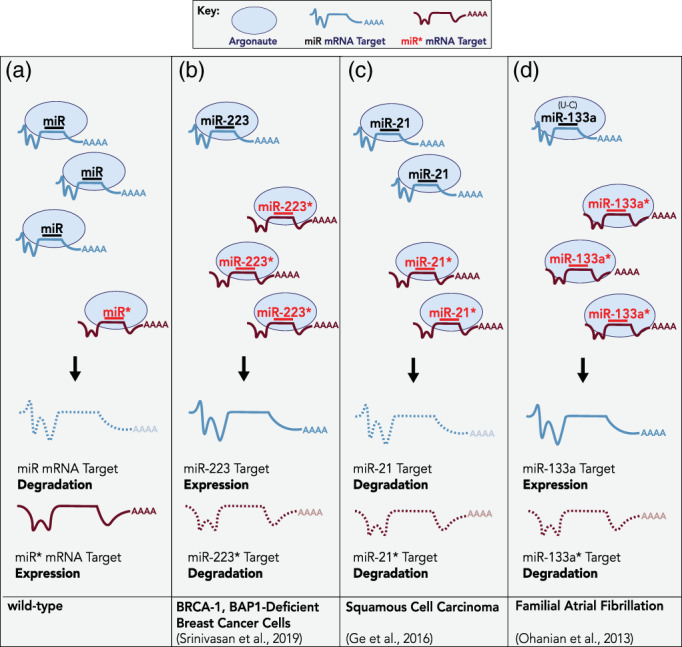
Examples of dysregulated miRNA strand selection in human disease. (a) Under wild‐type conditions, the miRNA guide strand is selected and loaded into miRISC in a preferential fashion. As the passenger strand is degraded, mRNAs matching the seed of the miR* strand are maintained in an active state whereas seed‐matched targets of guide‐loaded miRISC are repressed. (b) In BRCA1‐BAP1 deficient breast cancer cells, which cannot perform homologous recombination, miR‐223 arm switching leads to down‐regulation of miR‐223* targets and expression of miR‐233 mRNA targets (Srinivasan et al., 2019). (c) In squamous cell carcinoma (SCC), miR‐21* is upregulated and both miR‐21 and miR‐21* are loaded into miRISC and independently repress mRNAs that contribute to SCC pathogenesis (Ge et al., 2016). (d) A point mutation in miR‐133 (U‐C) associated with human atrial fibrillation alters the relative thermodynamic stability of the miR‐133 duplex ends and leads to selection of miR‐133* (Ohanian, Humphreys, Anderson, Preiss, & Fatkin, 2013). Presumably, altered mRNA target specificity by miR‐133* contributes to atrial fibrillation associated with this variation

There are many examples and several reported mechanisms by which de‐regulation of miRNA strand selection becomes associated with human disease. While under wild‐type conditions, most miRNAs have a dominantly expressed miRNA strand (Figure 6a), numerous miRNAs undergo arm switching in cancers (Chen et al., 2018). Cancer cells lacking BRCA1 (breast cancer 1) and BAP1 (BRCA1‐associated protein), which are deficient in homologous recombination, aberrantly express high levels of miR‐223* (5p) (Srinivasan et al., 2019; Figure 6b, Table 1). miR‐223 (3p) directly targets components of the nonhomologous end‐joining (NHEJ) pathway. Reduction of miR‐223 (3p) abundance and increase in miR‐223* (5p) stability, is proposed to de‐repress NHEJ and allow for cell proliferation (Srinivasan et al., 2019). Consistently, restoration of miR‐223 (3p) in BRCA1, BAP1‐deficient cancer cells is synthetic lethal, probably due to repression of NHEJ components (Srinivasan et al., 2019). Similarly, the strand bias of miR‐193a appears to be altered in breast cancer cell lines, as well as patient tissues (Tsai et al., 2016). In humans, miR‐193a locus produces two near equally abundant miRNAs: miR‐193a‐5p and miR‐193‐3p. The abundance of miR‐193a‐5p, but not miR‐193‐3p is significantly decreased in breast cancer and is believed to be due to reduced availability of miR‐193a‐5p targets (Tsai et al., 2016).

**TABLE 1:** Selected examples of altered miRNA abundances in human disease

Disease	miRNA	Strand abundance		Reference
		miR	miR*	
Breast cancer	miR‐223	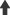	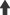	Sririvasan et al., 2019
	miR‐193a	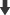 ^a^	n/a	Tsai et al., 2016
	miR‐24‐2	**=**	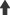	Martin et al., 2012
Papillary thyroid carcinoma	miR‐146a	n/a	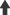 isomiRs	Jazdzewski et al., 2009
Multiple sclerosis	miR‐155	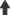	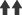	Mycko, Cichalewska, Cwiklinska, & Selmaj, 2015
Lung cancer	miR‐144	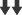	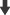	Song et al., 2018
	miR‐193b	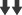	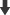	Choi, Shin, Lee, Ji, & Kim, 2019
Gastric cancer	miR‐574	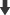	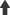	Zhang et al., 2019
Squamous cell carcinoma	miR‐21	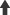	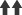	Ge, Zhang, Nikolova, Reva, & Fuchs, 2015
Atrial fibrillation	miR‐133	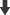	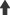	Ohanian et al., 2013
Glioblastoma	miR‐324	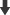	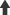	Kim et al., 2020

Abbreviations: =, unchanged; n/a, not assessed.

^a^ miR‐193‐5p is decreased (both arms highly abundant).

In other examples, a G/C polymorphism in the miR‐146a locus, associated with papillary thyroid carcinoma (PTC) progression, alters the sequence of miR‐146a* (3p) strand, and likely leads to changes in target repression, contributing to PTC (Jazdzewski et al., 2009; Labbaye & Testa, 2012), (Table 1). In animal models of multiple sclerosis, miR‐155* (3p) is highly up‐regulated in T‐helper (Th) cells, and promotes Th17 cell differentiation (Mycko et al., 2015). miR‐155* (3p) targets the Hsp40‐family genes Dnaja2 and Dnajb1, whose down‐regulation appears to directly contribute to the disease pathogenesis (Mycko et al., 2015), (Table 1). The miR‐24‐2* (5p) strand is upregulated in MCF‐7 breast cancer cell lines compared to control mammary epithelial cells (Martin et al., 2012), (Table 1). Here, miR‐24‐2* (5p) directly targets PKCα (protein kinase C), a factor that is required for the survival of MCF‐7 breast cancer cells (Martin et al., 2012; Weldon et al., 2005).

miR‐144* (5p) expression is down‐regulated in nonsmall cell lung cancer (NSCLC) (Song et al., 2018), (Table 1). miR‐144* (5p) directly targets ATF2, and it is possible that increased ATF2 levels upon reduced miR‐144* (5p) activity contributes to progression of NSCLC (Song et al., 2018). In another example, both miR‐193b (3p) and miR‐193b* (5p) are down regulated in lung cancer and restoring the levels of either strand suppresses malignant phenotypes including metastatic potential and cell proliferation (Choi et al., 2019), (Table 1). Both strands appear to target Cyclin‐D (CCND1), Ajuba LIM protein (AJUBA) and heart developmental protein with EGF like domains (HEG1) and knocking down those targets phenocopies restored miR‐193b levels (Choi et al., 2019). miR‐574* (5p) is oncogenic in colorectal cancer, NSCLC, and PTC (Cui, Tang, Chen, & Wang, 2014; Ji et al., 2012; Ma et al., 2016; X. Wang, Lu, Geng, Yang, & Shi, 2017; Zhou et al., 2015; Zhou et al., 2016), while miR‐574 (3p) is tumor suppressive in breast cancer and leukemia (Chiyomaru et al., 2013; Ujihira et al., 2015; Xu et al., 2020; Yao, Wu, Lindner, & Fox, 2017). In gastric cancer, both arms of miR‐574, miR‐219 and miR‐369 are readily detectable (Zhang et al., 2019), (Table 1). High expression of miR‐574* (5p) is associated with advanced gastric cancer stages (Zhang et al., 2019). Overexpression of miR‐574 *(5p) enhances cell proliferation while expression of miR‐574 (3p) suppresses proliferation. miR‐574* (5p) targets QKI6 (Quaking), while miR‐574‐3p targets ACVR1B (activin receptor type‐1), and depleting one miRNA strand's target phenocopies the loss of the other miRNA strand in gastric cancer cells (Zhang et al., 2019). Interestingly, over one‐third of the genes regulated by miR‐574‐3p and miR‐574‐5p overlap, highlighting that although each strand contains a unique seed sequence, there can be substantial shared targets of the two strands (Zhang et al., 2019).

In squamous cell carcinoma, both miR‐21 (5p) and miR‐21* (3p) are up‐regulated and each is required for cell survival (Ge et al., 2015; Figure 6c, Table 1). The oncogenicity of miR‐21* (3p) is mediated via its target gene, phosphatase, and actin regulator 4 (PPACTR4; Ge et al., 2015). miR‐21* (3p) downregulation of PPACTR4 appears to decrease protein phosphatase 1 (PP1) activity leading to increased phosphorylation and inactivation of the tumor suppressor Rb/E2F (retinoblastoma/E2 transcription factor), ultimately resulting in tumorigenesis (Ge et al., 2015; Ohanian et al., 2013). In another example, a single point mutation (U–C) that affects the relative TS of the human miR‐133a duplex results in altered 5p/3p ratios of miR‐133a and is associated with atrial fibrillation presumably through effects on mRNA targeting (Ohanian et al., 2013; Figure 6d).

Recently, uridylation of miR‐324 by TUT‐4/7 was shown to directly influence the arm switching of miR‐324 (Kim et al., 2020, Figure 4c, Table 1). TUT‐4/7 uridylate the 3p arm of miR‐324 leading to selection of the miR‐324* (3p) strand instead of the miR‐324 (5p) strand. Intriguingly, TUT‐4/7 are up‐regulated in glioblastoma and miR‐324 arm switching appears to correlate with a poor prognosis (Kim et al., 2020). Collectively, these data highlight that miRNA strand selection can be highly regulated and alterations to this normally robust process can have dramatic consequences and are prevalent in human disease.

## 6 SUMMARY AND PERSPECTIVES

**FIGURE 7:**
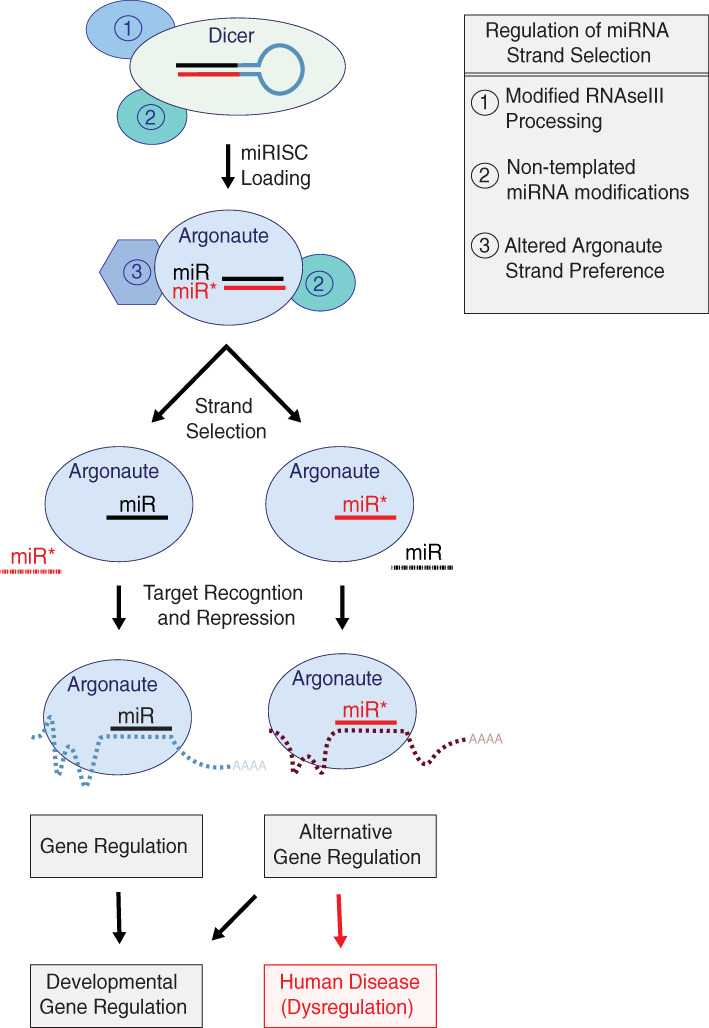
Summary of miRNA strand selection and regulatory mechanisms. Three major points of regulation for miRNA strand selection (1) altered miRNA processing by the Microprocessor complex (not shown) or Dicer, (2) remodeling of the miRNA duplex by nontemplated RNA modifications such as uridylation or A‐to‐I editing, and (3) changes in the strand preference of Argonaute. Once presented with a miRNA duplex, Argonaute makes a binary choice to load one miRNA strand and discard the other strand from miRISC. Alternate Argonaute programming leads to shifts in the target profile of miRISC based on the seed sequence of the loaded strand and is sometimes associated with human diseases

miRNA strand selection as part of miRISC programming is a critical event that ultimately dictates target gene identity. When regulated, it provides a mechanism to dramatically alter broad suites of distinct target genes. Strand selection appears to be mainly driven by two duplex‐intrinsic rules: miRNA 5′ nucleotide identity and the relative TS of duplex ends. Both miRNA strands can be functional, with arm switching playing a role during development through tissue‐specific or perhaps temporally‐regulated miRNA strand selection. Similarly, altered miRNA strand selection is associated with disease, presumably due to targeting distinct sets of mRNAs (Figure 7). While data suggest that Argonaute proteins contribute to miRNA strand selection by recognizing both the 5′ nucleotide and relative TS of miRNA strands, it has become increasingly clear that these rules can be “circumvented” under different physiological or pathological conditions, primarily through duplex sequence alterations or isomiR production.

There are at least three known regulatory points for miRNA strand selection: (a) altered processing of the miRNAs by RNAse III endonucleases generating templated isomiRs, (b) nontemplated miRNA isomiRs generated through deamination (A‐I editing) or uridylation, which remodel the miRNA duplex, and (c) altered strand preference of Argonaute (Figure 7). Notable is a recent study providing a direct molecular mechanism for miR‐324 arm switching through uridylation and subsequent alternative Dicer processing (Kim et al., 2020). Understanding how broadly this mechanism applies to all miRNAs will be an exciting avenue for future investigations. Potential regulatory points for miRNA strand selection include adenylation. While adenylation has not, to our knowledge, been demonstrated to play a role in miRNA strand selection, it is easy to imagine that adenylation could alter the TS of duplex ends and, therefore, strand selection. In addition, we can speculate that adenylation may result in alternative Dicer processing, similar to uridylation (Kim et al., 2020). Likewise, what roles Argonaute post‐translational modifications and interactions with regulatory factors may play in strand‐specific miRISC programming remain an intriguing question.

In vitro experiments have provided and will continue to provide important advances in understanding small RNA loading. However, the rules governing miRNA strand selection were determined using a limited number of purified components; whether these rules are sufficient to drive strand selection in vivo has not been extensively explored. At first glance, a significant number of miRNAs do not follow either the 5′ nucleotide or TS rules (see Figure 3). However, the analysis is limited, using the most highly expressed 5p and 3p strands for a given miRNA without taking isomiRs into consideration. While technically challenging, identification of duplexes from which the individual miRNA strands are observed would be an important advance in understanding how miRNA strand selection is regulated in vivo. Finally, multiple mechanisms may be involved in regulation of miRNA strand selection, with combinations of molecular inputs dictating which miRNA becomes loaded and to what extent. Further studies aimed at addressing how miRNA strand selection is determined and regulated in vivo should provide valuable insights toward understanding how miRNA strand selection is de‐regulated in human disease.

## CONFLICT OF INTEREST

The authors have declared no conflicts of interest for this article.

## AUTHOR CONTRIBUTIONS

**Jeffrey Medley:** Formal analysis; visualization; writing‐original draft; writing‐review and editing. **Ganesh Panzade:** Data curation; writing‐review and editing. **Anna Zinovyeva:** Conceptualization; funding acquisition; resources; supervision; visualization; writing‐original draft; writing‐review and editing.

## RELATED WIREs ARTICLES

Anatomy of RISC: how do small RNAs and chaperones activate Argonaute proteins?

The organization and regulation of mRNA‐protein complexes
